# Neosalvarsan

**Published:** 1912-08

**Authors:** W. W. Quinton

**Affiliations:** Buffalo, N. Y.


### Neosalvarsan

### Review of Recent Literature

By W. W. QUINTON, M.D.
Buffalo, N. Y.

The latest product of the Erlich laboratories bears the number 914 and has been named Neosalvarsan.

According to Erlich it is formed through a condensation of formaldehyde sulphoxylate of sodium ($CH_2$ (OH) O. SO. Na.) with Salvarsan.

He claims for this preparation: High solubility; neutral reaction; low toxicity; as effective if not more so than Salvarsan and that it does not cause reaction symptoms e. g., diarrhoea and vomiting, even in large doses.

It is a yellowish powder easily soluble in water and is much less toxic in its effect than Salvarsan. 1.5 gm. of Neosalvarsan is equivalent to 1.0 gm. of Salvarsan. In spite of this however, it is claimed that the new preparation is much more active than the old.

To make a solution of Neosalvarsan it is necessary only to pour the contents of an ampoule into freshly distilled water (5-10 cc if the solution is desired for intramuscular injection) and shake gently a few times. Vigorous shaking should be avoided as it will cause oxidation. It is entirely unnecessary to neutralize this solution. The temperature of the vehicle should be the same as that of the room. The water must be made fresh on the day of the injection. It is first filtered, then distilled and finally sterilized and cooled to the desired temperature.

If an intravenous solution is desired, Schreiber recommends that saline solutions in concentration up to 0.4% be used, as cloudiness will result if a greater concentration is employed and Neosalvarsan also seems to be more toxic in high concentrations.

The dose for the average male is 1.5 gm.; for the female, 1.2 gm. However a primary dose of 0.9 gm. for the male and 0.75 gm. for the female is recommended, which may be increased after noting the effect. Children will bear 0.15 gm. and nurslings 0.05 gm.

Schreiber outlines the following routine method for its administration:

First day, 0.9 gm.; third day, 1.2 gm.; fifth day, 1.35 gm.; seventh day, 1.5 gm.

Rytina gives four injections of 0.9 gm. at weekly intervals.

By the use of this new preparation the most rapid results are obtained with the least discomfort to the patient; the local and general disturbances incident to the injection being very slight.

Neosalvarsan is much better adapted to intramuscular use than Salvarsan. The initial pain caused by the injection may be avoided by injecting 5cc of a 1-2 per cent. solution of Novocain. Leaving the needle in situ, the Neosalvarsan may be injected a few minutes later.

The same extreme care in the selection of cases should be observed as with the use of Salvarsan and in this connection it might be as well to call attention to the fact that primary excision of the initial lesion is still considered of some importance in the treatment.

